# Long‐read sequencing reveals widespread intragenic structural variants in a recent allopolyploid crop plant

**DOI:** 10.1111/pbi.13456

**Published:** 2020-09-06

**Authors:** Harmeet Singh Chawla, HueyTyng Lee, Iulian Gabur, Paul Vollrath, Suriya Tamilselvan‐Nattar‐Amutha, Christian Obermeier, Sarah V. Schiessl, Jia‐Ming Song, Kede Liu, Liang Guo, Isobel A. P. Parkin, Rod J. Snowdon

**Affiliations:** ^1^ Department of Plant Breeding Justus Liebig University Giessen Germany; ^2^ Department of Botany and Molecular Evolution Senckenberg Research Institute and Natural History Museum Frankfurt Frankfurt am Main Germany; ^3^ National Key Laboratory of Crop Genetic Improvement Huazhong Agricultural University Wuhan China; ^4^ Agriculture and Agri‐Food Canada Saskatoon SK Canada

**Keywords:** *Brassica napus*, polyploidy, genome rearrangement, presence–absence variants, PAV

## Abstract

Genome structural variation (SV) contributes strongly to trait variation in eukaryotic species and may have an even higher functional significance than single‐nucleotide polymorphism (SNP). In recent years, there have been a number of studies associating large chromosomal scale SV ranging from hundreds of kilobases all the way up to a few megabases to key agronomic traits in plant genomes. However, there have been little or no efforts towards cataloguing small‐ (30–10 000 bp) to mid‐scale (10 000–30 000 bp) SV and their impact on evolution and adaptation‐related traits in plants. This might be attributed to complex and highly duplicated nature of plant genomes, which makes them difficult to assess using high‐throughput genome screening methods. Here, we describe how long‐read sequencing technologies can overcome this problem, revealing a surprisingly high level of widespread, small‐ to mid‐scale SV in a major allopolyploid crop species, *Brassica napus*. We found that up to 10% of all genes were affected by small‐ to mid‐scale SV events. Nearly half of these SV events ranged between 100 bp and 1000 bp, which makes them challenging to detect using short‐read Illumina sequencing. Examples demonstrating the contribution of such SV towards eco‐geographical adaptation and disease resistance in oilseed rape suggest that revisiting complex plant genomes using medium‐coverage long‐read sequencing might reveal unexpected levels of functional gene variation, with major implications for trait regulation and crop improvement.

## Introduction

The recent allopolyploid species *Brassica napus* L. (oilseed rape/canola/kale/rutabaga; genome AACC, 2n = 38) rapidly emerged as a globally important crop. Genome assembly and resequencing of *B*.* napus* (Chalhoub *et al*., 2014) revealed a highly complex and strongly duplicated genome with an unexpected extent of segmental exchanges among homoeologous chromosomes. In synthetic *B*.* napus* accessions, genome structural variants frequently span whole chromosomes or chromosome arms (Chalhoub *et al*., 2014; Samans *et al*., 2017). Naturally formed *B*.* napus* also shows widespread homoeologous exchanges, with similar distribution patterns (Hurgobin *et al*., 2018; Samans *et al*., 2017), that apparently arose during the allopolyploidization process (Leflon *et al*., 2006; Nicolas *et al*., 2007; Szadkowski *et al*., 2010). The wide extent of segmental deletion/duplication events in both synthetic and natural *B. napus* has been confirmed using other genome‐wide analysis methods, for example visualization based on mRNAseq data (He *et al*., 2017) or deletion calling from SNP array data (Gabur *et al*., 2018; Grandke *et al*., 2016). Critically, numerous examples have connected genome SV in *B. napus* to important agronomic traits (Gabur *et al*., 2018; Gabur *et al*., 2019; Liu *et al*., 2012; Stein *et al*., 2017). These studies revealed the important role of SV in the creation of *de novo* variation for adaptation and breeding; however, the methods used were not yet capable of resolving SV at gene scale.

A first example of intragenic SV impacting quantitatively inherited traits in *B. napus* was reported by Qian *et al*. (2016), who demonstrated that deletion of exons 2 and 3 from a *B. napus* orthologue of Mendel’s 'Green Cotyledon' gene (the Staygreen gene *NON‐YELLOWING 1*; *NYE1*) associated with quantitative variation for chlorophyll and oil content. Unfortunately, such small deletions are challenging to reliably detect using short‐read sequencing or low‐cost marker arrays, so that their genome‐wide extent could not yet be investigated in detail. In this study, using *B. napus* as an example for a plant genome with widespread structural variation, we demonstrate the power of whole‐genome long‐read sequencing for high‐resolution detection of intragenic SV. The results reveal widespread functional variation on a completely unexpected scale, suggesting that small‐ to mid‐scale SV may be a major driver of functional gene diversity in this recent polyploid crop. With the growing accessibility, accuracy and cost‐effectiveness of long‐read sequencing, our results suggest that there could be enormous promise in revisiting complex crop genomes to discover potentially novel functional SV which has previously been overlooked.

## Results and discussions

### **Long‐read sequencing reveals novel SV diversity in** *B. napus*

We sequenced 4 *B. napus* accessions with long reads using the Oxford Nanopore Technology (ONT) and 8 additional accessions using the Pacific Biosciences (PacBio) platform (data from Song *et al*. (2020)). The genotype panel included three vernalization‐dependent winter‐type accessions, 3 vernalization‐independent spring‐type accessions, 4 semi‐winter accessions and 2 synthetic *B. napus* accessions (a winter‐type and a spring‐type). All accessions were sequenced to between ~ 30x and ~ 50x whole‐genome coverage (between 30 and 50 GB of data). Reads were aligned to the *B. napus* Darmor‐*bzh* version 4.1 reference genome (Chalhoub *et al*., 2014) using the long‐read aligner NGMLR (https://github.com/philres/ngmlr) (Sedlazeck *et al*., 2018) and called for genome‐wide SV using the SV‐calling algorithm Sniffles (Sedlazeck *et al*., 2018). N50 values ranging from 10 552 to 15 369 bp were obtained for the 8 PacBio datasets, while in the 4 ONT datasets the N50 ranged from 10 756 to 28 916 bp (Table 1, Table S1). After aligning to the Darmor‐*bzh* v4.1 reference genome, the total number of SV events called by Sniffles ranged from 51 463 to 108 335. PacBio and ONT sequencing can potentially result in systemic differences in SV calling because of their different error profiles. PacBio sequencing is known to be enriched for small insertion errors, whereas ONT suffers from deletions especially in homopolymer regions (Sedlazeck *et al*., 2018). To neutralize systematic bias due to different error profiles, we used different noise models for aligning ONT and PacBio datasets. For ONT datasets, we used the '‐x ont' flag for NGMLR, whereas this was omitted for PacBio datasets because NGMLR expects a PacBio dataset by default. To demonstrate that this flag was effective in expunging the majority of spurious SV calls, we compared variant calls between ONT and PacBio datasets from the same winter oilseed rape genotype, Express 617, using data used by Lee *et al*. (2020) to assemble the Express 617 genome. Overall, 27 106 and 33 424 quality‐filtered SV calls from respective ONT and PacBio libraries of Express 617 were merged using SURVIVOR, resulting in a combined set of 34 885 SV. We found 82.7 per cent (28 857) of the total SV to be supported by both ONT and PacBio reads, indicating that both long‐read technologies generate highly suitable data to accurately capture a majority of small‐ to mid‐scale genome‐wide SV events.

**Table 1:** Number and size distributions of SV detected in 12 *B. napus* genotypes

Genotype	Data type	Ecotype	N50 for raw reads	Quality‐filtered SV	Intragenic SV	Maximum size of SV	Median size SV
Express 617	ONT	Winter	10 756	27 106	5898	16 931	341
Quinta	PacBio	Winter	14 192	32 349	7085	15 869	353
Tapidor	PacBio	Winter	14 448	32 757	7291	15 289	344
ZS11	PacBio	Semi‐winter	10 552	37 496	9004	11 312	281
Zheyou7	PacBio	Semi‐winter	12 370	38 590	9042	17 001	305
Gangan	PacBio	Semi‐winter	14 064	35 560	8366	14 264	335
Shengli	PacBio	Semi‐winter	13 828	39 622	9501	12 207	321
PAK85912	ONT	Spring	28 916	23 177	5011	28 777	584
N99	ONT	Spring	27 139	34 848	7482	26 183	509
Westar	PacBio	Spring	13 810	37 138	8575	17 615	332
R53	ONT	Winter synthetic	11 253	33 851	8647	12 635	296
No2127	PacBio	Spring synthetic	15 369	44 516	10 675	15 565	304

ONT, Oxford Nanopore Technologies; PacBio, Pacific Biosciences; SV, structural variant.

To minimize false‐positive calls derived from reference mis‐assemblies, we followed a highly stringent quality‐filtering approach that removed 54.4–59.4% of the total predicted SV. This procedure resulted in a final set of 27 106 to 44 516 high‐quality SV events (Table 1). To evaluate the impact of assembly errors on SV‐calling rates, we compared results after aligning (using the same procedure) to a pseudo‐reference constructed by combining the high‐quality long‐read reference assemblies of *Brassica rapa* (A subgenome) and *Brassica oleracea* (C subgenome) published recently by Belser *et al*. (2018). Using this pseudo‐reference assembly, we detected between 41 436 and 50 907 quality‐filtered SV across the 12 *B. napus* genotypes. There are two possible explanations for the higher number of SV. Firstly, the pseudo‐reference assembly (957 Mbp) is nearly 10 per cent larger than the *B. napus* Darmor‐*bzh* v4.1 reference (849.7 Mbp). Secondly, SV detected using the pseudo‐reference assembly will also reflect genomic differences between the unknown diploid progenitors of *B. napus* and the two diploid genotypes from which this pseudo‐assembly was generated. To further validate our SV detection approach, we therefore compared the number of SV per megabase, detected using the two different genome assemblies for each of the 19 chromosomes across 12 genotypes. This showed a correspondence of 77.08 per cent, suggesting that the latter may be the predominant cause.

**Figure 1:**
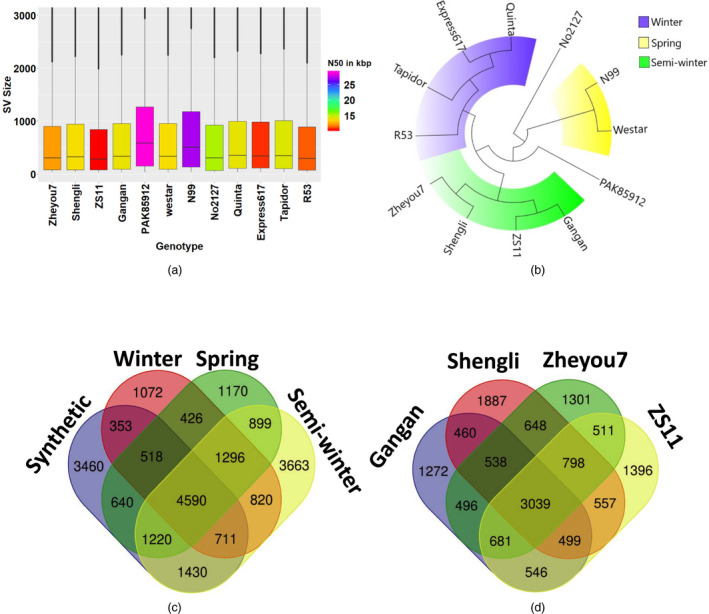
Gene scale SV in oilseed rape. (a) Box plots showing size distributions of SV events detected in 12 *B.napus* genotypes. (b) Maximum‐likelihood tree showing genetic relationships among 12 *B. napus* genotypes based solely on genome‐wide SV events, revealing clear clustering into the appropriate eco‐geographical morphotype groups. (c) Venn diagram showing the numbers of common or unique genes carrying intragenic SV events across three divergent ecotypes and synthetic *B. napus*, respectively. (d) Venn diagram representing the numbers of common or unique genes carrying intragenic SV events across four semi‐winter *B. napus* accessions.

After alignment to the Darmor‐*bzh* v4.1 reference genome, the median detected SV size across the 12 accessions ranged from 296 bp to 584 bp. The spring‐type accessions N99 and PAK85912 had the largest median SV size (509 and 584 bp, respectively), which might be attributable to the longer read lengths for these two genotypes (N50 = 27 139 bp and 28 916 bp, respectively) (Figure 1a). The largest SV event (28 777 bp) was also detected in the spring‐type accession PAK85912, suggesting that read length plays a critical role in the ability to detect large and complex SV events. A complete lack of SV calls on chromosome C02 of the winter oilseed rape accession R53, using both the pseudo‐reference and the Darmor‐*bzh* assembly. No SV calls were observed on chromosome C02 of the synthetic *B. napus* accession R53. This is because C02 is deleted in R53 and replaced by its homeolog A02, as reported previously by Stein *et al*. (2017). Around half of all detected, high‐confidence SV events (46.8 to 53.2 % across the 12 genotypes) ranged in size from ~ 100–1000 bp (Tables S2‐S3). These small SV represent a novel genetic diversity resource that was previously unnoticed due to the insufficient resolution of high‐throughput genotyping platforms such as SNP genotyping arrays and a very high false‐positive rates (up to 89%) of short‐read sequencing data (Mahmoud *et al*., 2019; Sedlazeck *et al*., 2018).

### Subgenomic differences in SV frequency

**Figure 2:**
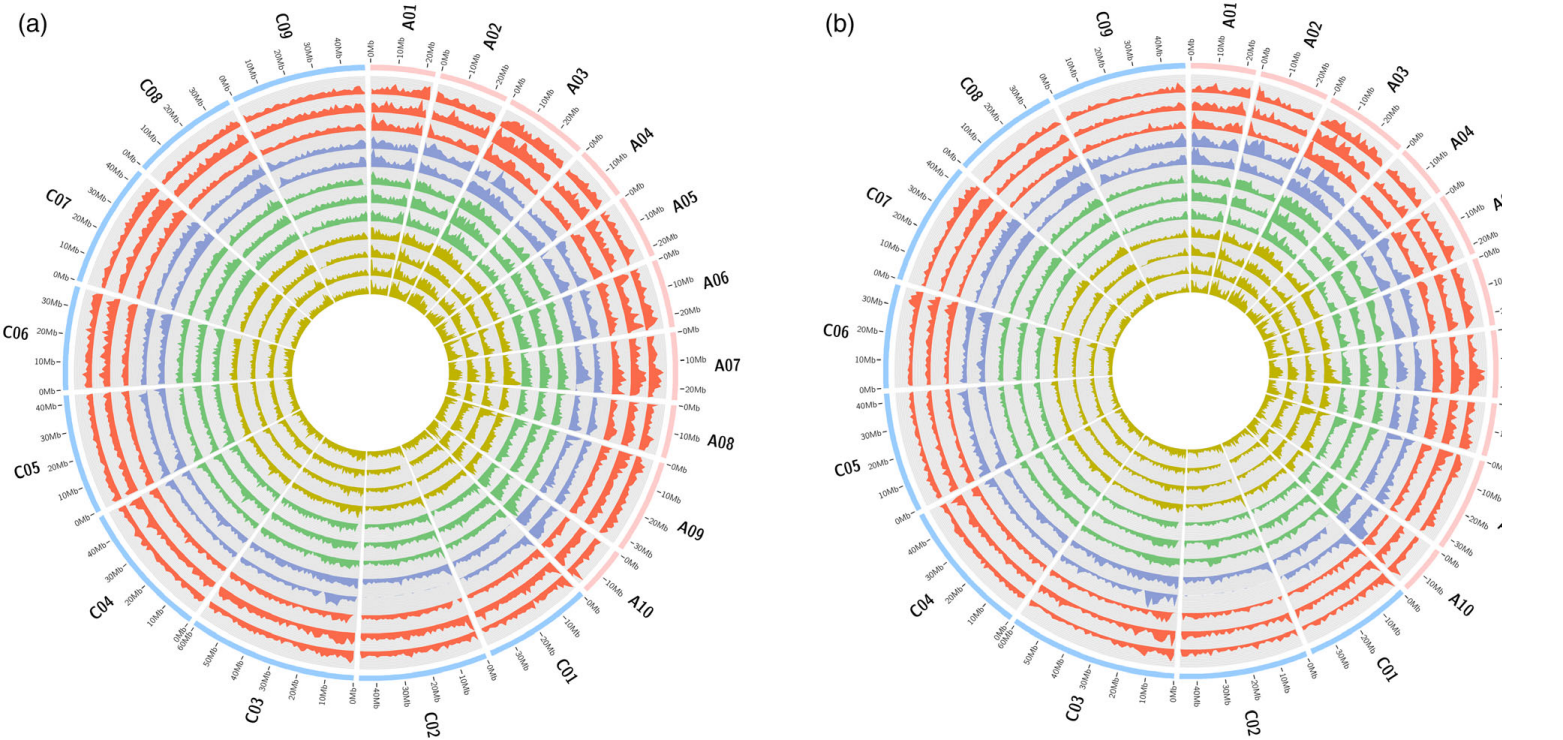
Genome‐wide distribution of small‐ to mid‐scale insertions and deletions in *B. napus*. (a) Circos plot depicting number of small to mid‐scale deletion events calculated in 1 Mbp windows across 19 chromosomes of 12 *B. napus* accessions. Each track represents a single genotype in the following order from outside to inside: Express 617, Quinta, Tapidor, R53, No2127, N99, Westar, PAK85912, Gangan, Shengli, Zheyou7 and ZS11. Colours of tracks represent different types of *B. napus*. The red, blue, green and yellow track colours represent winter‐type, synthetic, spring‐type and semi‐winter accessions, respectively. (b) Circos plot depicting the frequency of small to mid‐scale insertion events in 1 Mbp windows across 19 chromosomes of 12 *B. napus* genotypes. Each track represents a single genotype in the following order from outside to inside: Express 617, Quinta, Tapidor, R53, No2127, N99, Westar, PAK85912, Gangan, Shengli, Zheyou7 and ZS11. Colours of tracks represent different types of *B. napus*. The red, blue, green and yellow track colours represent winter‐type, synthetic, spring‐type and semi‐winter accessions, respectively.

Comparison of subgenomic SV frequency revealed significantly higher numbers of small‐ to mid‐scale SV per megabase in the *B. napus* A subgenome than the C subgenome in all twelve analysed genotypes (Figure 2a and b, Tables S4‐S5). This reflects a corresponding subgenomic bias also observed for large‐scale SV in *B. napus* (Samans *et al*., 2017), and this could also be attributable to repeated introgressions from the A genome of *B. rapa* during the breeding history of *B. napus* (Lu *et al*., 2019). Samans *et al*. (2017) reported a significant enrichment for large‐scale segmental deletions in the C‐subgenome of *B. napus* resulting from homoeologous exchanges. In contrast, we observed no bias for small to mid‐scale deletions in the C‐subgenome of the 12 sequenced *B. napus* accessions (Tables S6‐S7). This indicates that a different molecular mechanism may be responsible for the generation of large and small to mid‐scale SV events in the rapeseed genome. Unexpectedly, we found that between 5% (Express 617) and 10% (No2127) of all genes detected in the twelve accessions were affected by small to mid‐scale SV events. This represents a previously completely unknown extent of functional gene modification as a result of post‐polyploidization genome restructuring. It also underlines the massive selection potential arising from intergenomic disruption during the act of allopolyploidization (Nicolas *et al*., 2008; Nicolas *et al*., 2007; Szadkowski *et al*., 2010), and the great significance of post‐polyploidization intergenomic restructuring for polyploid crop evolution (Samans *et al*., 2017).

### Small‐ to mid‐scale SV underlining eco‐geographical differentiation in *B. napus*

**Figure 3:**
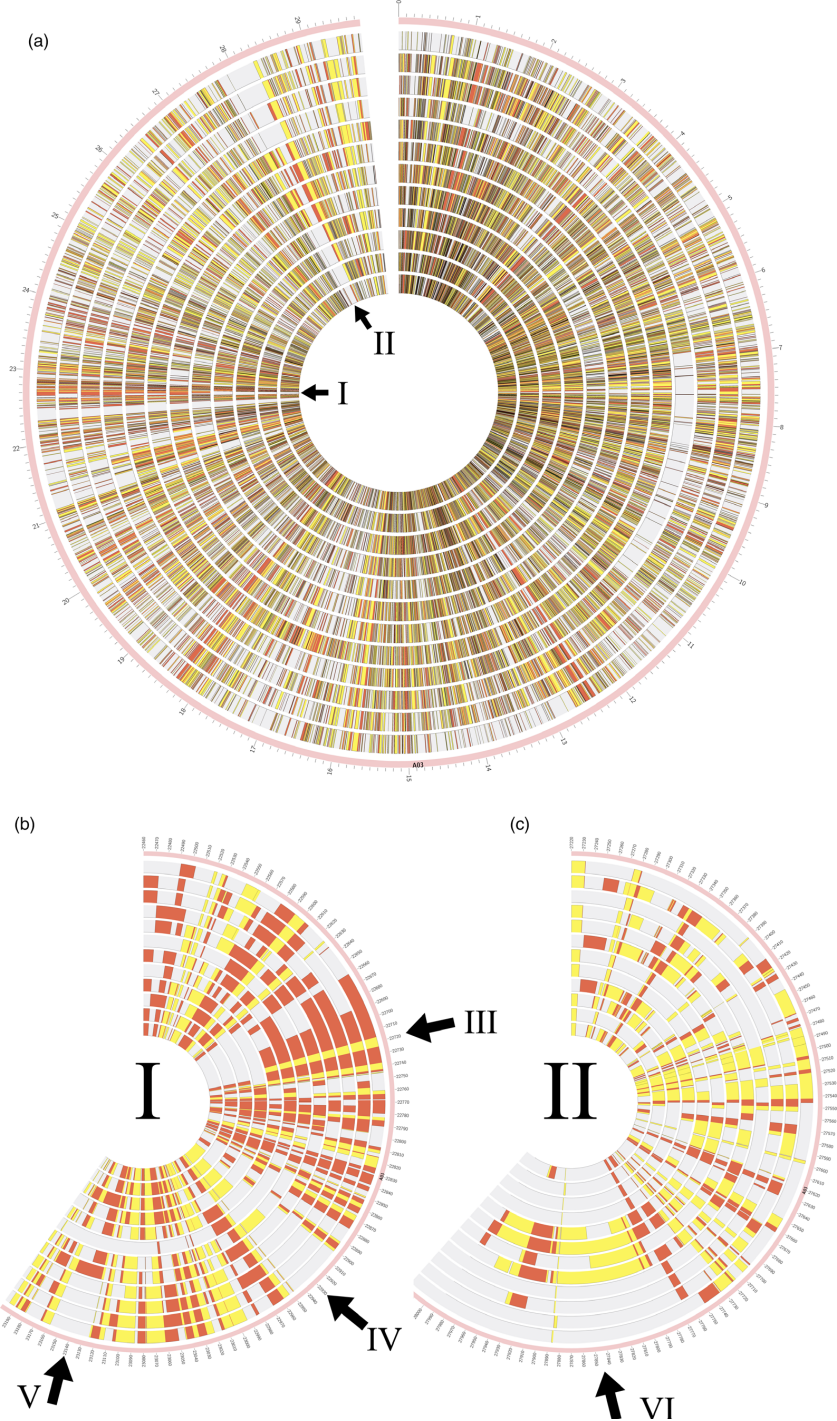
Small‐ to mid‐scale genomic rearrangements on chromosome A03 of *B.napus*. (a) Circos plot showing small‐ to mid‐scale insertion and deletion events in 12 *B. napus* accessions, using chromosome A03 as an example. Each track represents a single accession in the following order from outside to inside: Express 617, Quinta, Tapidor, R53 (all winter type), No2127, N99, Westar, PAK85912 (spring type), Gangan, Shengli, Zheyou7 and ZS11 (semi‐winter type). Deletions are represented by yellow blocks, whereas insertions are shown by red blocks. Darker blocks in (A) represent regions containing both deletions and insertions in different genotypes. Arrows I and II mark selected segmental SV events specific for a particular ecotype. (b) Expanded view of the chromosome segment depicted by arrow I in A. Arrow III represents a 50 kbp region containing segmental deletion and insertion events detected in all winter and spring ecotypes but not in the semi‐winter types. Arrow IV indicates a 40 kbp region containing segmental deletions detected only in the four semi‐winter types and three of spring types. Arrow V indicates a 40 kbp region containing segmental insertions detected only in the four semi‐winter types and one of the spring types. (c) Expanded view of the chromosome segment depicted by arrow II in A. Arrow VI indicates a 120 kbp region containing segmental insertions only in the four spring types.

As expected, strong SV differentiation from the winter‐type oilseed reference genotype Darmor‐*bzh* was found in the divergent semi‐winter and spring ecotypes, and in genetically distant synthetic *B. napus* accessions R53 and No2127 (Figure 3). Unexpectedly, however, the winter‐type accessions Express 617, Tapidor and Quinta also showed high levels of SV compared to Darmor‐*bzh*, despite a related breeding history and partially shared pedigree (e.g. Express 617). According to (Lu *et al*., 2019), who used whole‐genome resequencing data to investigate the species origin and evolution of *B. napus*, spring and semi‐winter types arose only very recently (<500 years) from winter types. Our data concur with this assumption, with fewer genes carrying SV in winter‐type accessions (1072) than in spring (1170) or semi‐winter (3663) ecotypes (Figure 1c). Furthermore, we also detected small‐ to mid‐scale SV within each ecotype; for example, 1272–1887 genes carrying unique SV events were found among the four semi‐winter accessions (Figure 1d). The unexpectedly high structural gene diversification both between and within ecotypes suggests that *de novo* generation of small‐ to mid‐scale SV may also be ongoing in recent breeding history. Overall, 4590 of the called intragenic SV were common among the four *B. napus* forms, indicating putative SV events specific to Darmor‐*bzh*. These could possibly be attributed to errors in the Darmor‐*bzh* reference assembly; however, the similar number of unique intragenic SV detected only in semi‐winter types (3663) suggests that this frequency is not unexpected in the context of the other results. Repeating the analysis with the concatenated pseudo‐reference from *B. rapa* plus *B. oleracea* gave comparable results (6248 common among all sequenced *B. napus* forms, 2919 unique to semi‐winter ecotypes).

To evaluate the influence of SV on eco‐geographical adaptation and potential species diversification, we constructed a maximum‐likelihood (ML) tree for the 12 *B. napus* lines based solely on SV detected using long‐read sequencing data. The resulting tree (Figure 1b) comprised 3 divergent clades representing 3 ecotypes of *B. napus* (winter, semi‐winter and spring). In contrast with genetic clustering based on genome‐wide SNP data, which reveals high sequence diversification between synthetic and natural *B. napus* (Bus *et al*., 2011), the two synthetic accessions R53 and No2127 did not fall into separate clades. Instead, the winter‐type R53 clustered closest together with the natural winter‐type accessions and the spring‐type No2127 clustered with the natural spring‐type accessions. This suggests that small‐ to mid‐size SV events originating during or immediately after allopolyploidization might rapidly confer eco‐geographical adaptation. Although hundreds to thousands of genes carrying unique SV events were detected in each individual accession, the intriguing observation that their cumulative clustering reflects eco‐geographical adaptation forms suggests a possible key role of SV in rapid functional adaptation. Overall, the distribution and frequency of SV events in all investigated accessions suggest that small‐ to mid‐scale SV may be a major, previously unknown source of functional genetic variation in *B. napus*.

Unfortunately, a catalogued and validated 'truth set' of genomic SV is not yet established for *B. napus* or other complex plant genomes. This makes it crucial to validate SV predicted from long reads using independent validation methods. On the other hand, manual verification of thousands of SV events (for example using PCR) is not realistic. To obtain first insight into the validity of the SV called using our pipeline, we selected relevant potentially functional examples representing possible functional mutations in flowering‐time and disease resistance‐related genes. We validated the detected SV events using different independent methods in a total of 4 *B. napus* genotypes including two springs, one winter and a synthetic.

### Small‐ to mid‐scale SV events impact *B. napus* flowering‐time pathway genes

**Figure 4:**
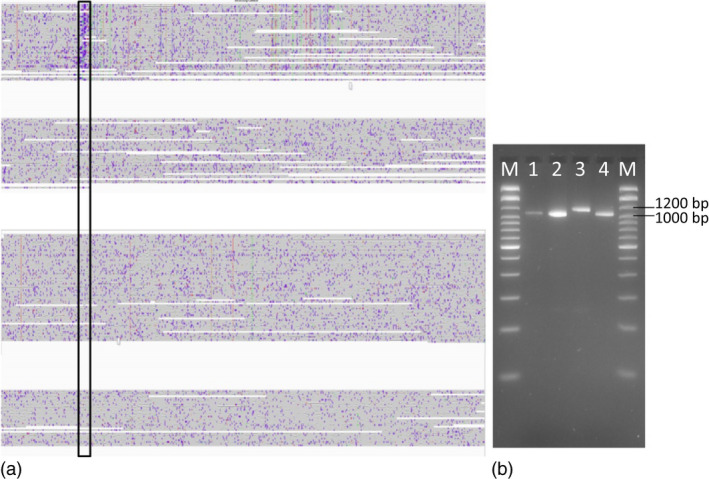
Small‐scale SV in a key flowering‐time gene *Vernalization Insensitive 3* on chromosome C03 (*BnVIN3.C03*). (a) 90 bp insertion detected only in accession Express 617 (highlighted in the black box) in an orthologue *BnVIN3.C03* was revealed by aligning ONT reads from 4 different genotypes to the Darmor*‐bzh *reference* *version 4.1. (b) Agarose gel image of PCR products spanning the insertion polymorphism. M:100 bp ladder; 1: N99; 2: PAK85912; 3: Express 617; 4: R53. As expected, Express 617 exhibits 1170 bp PCR product, whereas the other three genotypes all show a 1060 bp amplicon.

In order to understand the impact of gene scale rearrangements on eco‐geographical adaptations in *B. napus*, we examined the abundance of SV in the known *B. napus* orthologs of all known genes from the *Arabidopsis* flowering‐time pathway. Whereas most of these genes are present in only a single copy in *Arabidopsis*, all have multiple duplicates in *B. napus* (Schiessl *et al*., 2014). Although many *B. napus* flowering‐time gene orthologues are known to be affected by copy‐number variation (CNV), the exact positions of copy‐number variants and other small‐ to mid‐scale forms of SV could not be determined from previous short‐read resequencing data (Schiessl *et al*., 2017). In this study, we used the long‐read data from two winter and two spring oilseed rape accessions to identify CNV in the form of duplications within flowering‐time genes (Tables S8 and S9). Only 3 to 4 per cent of all genes in the flowering‐time pathway showed CNV in these genotypes, whereas 24.7 % (44 of 178), including numerous key regulatory genes, contained one or more small to mid‐scale insertions or deletions. Therefore, we did not perform CNV analysis in the remaining 8 genotypes and focused instead on small‐ to mid‐scale SV in the full dataset. For example, we detected a 90 bp insertion in an orthologue of *Vernalization Insensitive 3* on chromosome C03 (*BnVIN3*.*C03*, *BnaC03g12980D*) in 3 out of 12 total genotypes, Express 617, No2127 and Zheyou7 (Figure 4a). Successful validation of this insertion via PCR, using primers designed from the SV‐flanking sequences, is shown in Figure 4b. The same insertion was undetectable using only the short‐read sequence‐capture data of Schiessl *et al*. (2017). In two out of three spring accessions, N99 and PAK85912, we detected a 2.8 kbp insertion in a *B. napus* orthologue of the key vernalization regulator *Flowering Locus C* (*BnFLC.A02, BnaA02g00370D*), a variant previously reported by Chen *et al*. (2018) to be causal for early flowering (Appendix S1).

**Figure 5:**
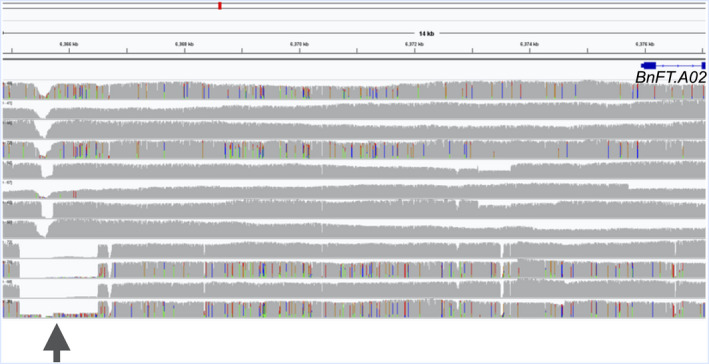
1.3 kbp deletion in the putative promoter of *BnFT.A02* (*BnaA02g12130D*). Each track represents a single genotype in the following order from top to bottom: Express 617, Tapidor, Quinta, R53, Shengli, ZS11, Gangan, Zheyou7, No2127, N99, Westar and PAK85912. The arrow indicates a 1.3 kbp deletion in putative promoter region in *BnFT.A02* (*BnaA02g12130D*) for all four spring accessions (No2127, N99, Westar and PAK85912).

In a second case study, we analysed SV events in key vernalization genes that differentiate between the vernalization‐dependent and vernalization‐independent *B. napus* accessions in our panel. A number of interesting, putative functional variants were detected. For example, we detected a 1.3 kbp deletion (Figure 5 **)** in the putative promoter of *BnFT.A02* (*BnaA02g12130D*), located between 6 365 143 and 6 366 504 bp on chromosome A02. This deletion was exclusively detected in all 4 spring accessions. *BnFT.A02* has been reported to be differentially expressed among winter, spring and semi‐winter type *B. napus* by Wu *et al*. (2019) and the 1.3 kbp deletion in its promoter region might explain the cause for this differential expression. To further validate our hypothesis that this 1.3 kbp deletion in *BnFT.A02* associates with vernalization behaviour of oilseed rape, we genotyped it using a locus‐specific PCR assay in 25 vernalization‐dependent and 25 vernalization‐independent accessions from the ERANET‐ASSYST *B. napus* diversity set (Bus *et al*., 2011) (Table S10). Eighty per cent of the vernalization‐independent oilseed rape accessions were found to contain the 1.3 kbp deletion in *BnFT.A02*, whereas a majority of vernalization‐dependent winter types (79 per cent) showed no deletion. The strong co‐segregation of this SV with vernalization behaviour might indicate a potentially crucial role for this genomic rearrangement in eco‐geographical adaptation.

### Intragenic SV events associated with disease resistance in oilseed rape

**Figure 6:**
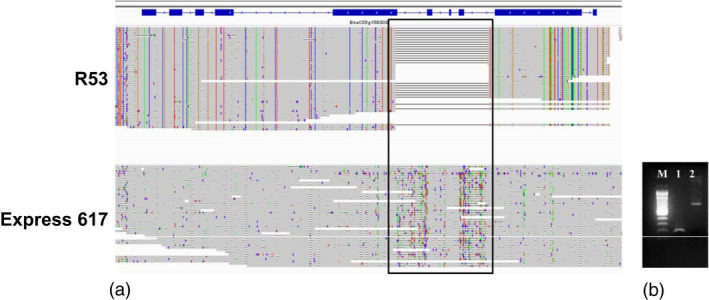
SV contributing to disease resistance. (a) 700 bp deletion (highlighted in the black box) was detected in accession R53 that caused the loss of three exons of a *4‐Coumarate:CoA Ligase* (*4CL*) gene *(BnaC05g15830D)*. (b) Agarose gel image of PCR product from the same deletion. M: 100 bp ladder; 1: PCR product originating from R53; 2: product originating from Express 617. As expected, Express 617 exhibits a 900 bp PCR product, whereas R53 shows a 200bp amplicon.

Samans *et al*. (2017) and Hurgobin *et al*. (2018) revealed that defence‐related R‐genes involved in monogenic resistance are particularly enriched in genome regions affected by large‐scale SV in *B. napus*. Gabur *et al*. (2020) reported gene presence–absence variations (PAV) in 23 to 51% genes within confidence intervals of QTL for *V. longisporum* resistance in *B. napus*. In a third case study related to a prominent disease resistance in oilseed rape, we investigated the impact of SV in resistance‐related genes co‐localizing with QTL for quantitative disease resistance in a bi‐parental cross between the sequenced accessions Express 617 and R53. These two accessions differ strongly in their resistance reaction to the important fungal pathogen *V longisporum* (Obermeier *et al*., 2013), and SV detected between the two parental lines were selected for validation based on their co‐localization to resistance‐related genes in corresponding resistance QTL (see Methods for selection criteria for PCR validation of SV events). Most interestingly, we identified a 700 bp deletion in R53 that caused the loss of three exons of a *4‐Coumarate:CoA Ligase* (*4CL*) gene (*BnaC05g15830D*). In the genetic map from the Express 617 × R53 mapping population, this gene is located within a major QTL for *V. longisporum* resistance on *B. napus* chromosome C05 (Obermeier *et al*., 2013). 4CL is a critical enzyme involved in the phenylpropanoid pathway (Li *et al*., 2015) and Obermeier *et al*. (2013) reported that major QTL for phenylpropanoid compounds co‐localized with the QTL for *V. longisporum* resistance in the Express 617 × R53 mapping population. Locus‐specific PCR primers, spanning the putative SV predicted by the long sequence reads, amplified 900 bp and 200 bp fragments for Express 617 and R53, respectively (Figure 6a,b), confirming the expected 700 bp deletion. Re‐screening of the PCR markers for the 700 bp deletion in the doubled haploid mapping population from Express 617 × R53 confirmed their co‐localization with the QTL and a strong effect on resistance of up to *R* ^2^ = 19.4%.

### Implications of long‐read sequencing technologies for discovery of functional diversity

Of nine additional SV events we evaluated using PCR, all showed the expected PCR products corresponding to the deletions or insertions predicted by the long‐read SV calling. These results underline the apparent effectiveness of long sequence reads for accurately detecting and anchoring insertions/deletions in a broad size range from under 100 bp up to multiple kbp. In contrast, Illumina short reads from regions corresponding to insertions not present in available reference genomes remain un‐aligned in alignment‐based resequencing approaches, meaning that their genomic localization using short‐read data can be achieved only by whole‐genome *de novo* assembly. Our results in *B. napus* showed that *de novo* SV events appear to occur at an unexpectedly high rate. Depending on the genotype, 49–75% of intragenic SV events were located in exons in relation to introns (Table S11), indicating high potential for functional implications. Given the very high rate and the differences among genotypes, it remains unclear how many high‐quality reference genomes will be necessary to construct a representative pangenome that captures the majority of the genome‐wide functional SV landscape.

This study provides one of the very first insights into genome‐wide, gene scale SV linked to important agronomic traits in a major crop species. Recently, Yang et al. (2019) revealed a similar scale of widespread SV by comparing whole‐genome assemblies of two diverse maize accessions. However, the cost of genome assembly is still much too high to capture the full extent of species‐wide SV in large numbers of genotypes, particularly in species like *B. napus* with dynamic polyploid genomes in which genome rearrangement may even still be ongoing. Our successful verification of 10 out of 10 SV selected events via PCR (Table S12) gives us high confidence that SV predicted using medium‐coverage long‐read data with our calling strategy are genuine. This provides a relatively cost‐effective method to assay larger germplasm collections without ascertainment bias.

The occurrence of SV events in a size range corresponding to intragenic rearrangements (~100–1000 nt) has been ignored in most crop species in the past, due to the limited resolution of short‐read resequencing. Although presence–absence calling from genome‐wide SNP array data has been successful in isolated cases in establishing QTL associations (e.g. Gabur et al., 2018a), SNP‐based genome‐wide association (GWAS) studies are unable to tag causative SV in crops and genome regions in which high levels of LD decay surround the SV events (Zhou *et al*., 2019). Array‐based approaches to call PAV or homoeologous exchanges (e.g. Grandke *et al*., 2016) are therefore likely to ignore potentially functional SV events. Reduced costs, considerably improved read accuracy and significantly increased average read lengths today make long‐read sequencing technologies a viable option not only for accurate assemblies of complex plant genomes (Belser *et al*., 2018), but increasingly also for genome‐wide resequencing. Our results suggest that simple reference‐based resequencing and alignment with long reads can uncover a new dimension of genetic and genomic diversity associated with important traits in crop plants. Particularly in polyploid plants (Schiessl *et al*., 2019), this may lead to discovery of previously unknown levels of functional diversity of major interest for breeding and crop adaptation.

## Experimental procedures

### Plant material

We chose 12 *B. napus* genotypes (Table 1) comprising of three winter, four semi‐winter, three spring and two synthetics (one each of winter and spring).

### DNA isolation for Oxford Nanopore Technology (ONT) sequencing

High‐molecular‐weight DNA was isolated using DNA isolation protocol modified from Mayjonade *et al*. (2016). Young leaves were harvested from rapeseed plants at 4–6 leaf stage and flash frozen using liquid nitrogen. Frozen leaf material was ground to fine powder using a mortar and pestle and transferred to 15 mL Falcon tube. A total of 4–5 mL of pre‐heated lysis buffer (1% w/v PVP40, 1% w/v PVP10, 500 mm NaCl, 100 mm TRIS pH8, 50 mm EDTA, 1.25% w/v SDS, 1% (w/v) Na_2_S_2_O_5_, 5 mm C_4_H_10_O_2_S_2_, 1 % v/v Triton X‐100) was added in order to disrupt the cell wall. The lysate was incubated for 30 min at 37°C in a thermomixer. A total of 0.3 volumes of 5M potassium acetate was added to the lysate and spun at 8000g for 12 min at 4°C to precipitates sodium dodecyl sulphate (SDS) and SDS‐bound proteins in order to obtain clean DNA. Finally, magnetic beads were used to recover cleaned DNA.

### Library preparation for ONT sequencing

Between 1–3 ug of DNA was used to prepare the sequencing library, using the ligation sequencing kit SQK‐LSK108 or SQK‐LSK109 according to the manufacturer’s recommendations. Genomic DNA was subjected to end repair followed by a bead clean‐up. Sequencing adaptors were then ligated to the end‐repaired DNA. Finally, the adaptor ligated DNA was once again subjected to bead cleaning. DNA was finally loaded onto an Oxford Nanopore MinION flow cell for sequencing.

### Pacific biosciences (PacBio) sequencing

Raw PacBio reads originating from 8 genotypes (Quinta, Tapidor, No2127, Westar, Gangan, Shengli, Zheyou7 and ZS11) were downloaded from NCBI short‐read archive (Accession number PRJNA546246) with the permission from the authors.

### Bioinformatics analysis

#### Alignment and SV calling for ONT data

Raw fast5 files obtained by the MinION device were base‐called using ONT provided base‐caller, Albacore. Raw uncorrected reads from various flow cells were combined into single fastq file for each genotype. This fastq file was used to align the Nanopore reads to the publically available *B. napus* reference genome assembly Darmor‐bzh v4.1 (Chalhoub *et al*., 2014) and also to a concatenated pseudo‐reference assembly comprising the *B. rapa* and *B. oleracea* reference assemblies recently published by Belser *et al*. (2018), using NGMLR version 0.2.7 (Sedlazeck *et al*., 2018) with default settings except for '‐x ont' flag, representing parameter presets for ONT. NGMLR produced an un‐sorted SAM file as an output, which was converted to a sorted BAM file using Samtools version 1.9 (Li *et al*., 2009). Genomic variants were called using Sniffles version 1.0.10 (Sedlazeck *et al*., 2018) using the preset parameters.

#### Alignment and SV calling for PacBio data

Since 8 PacBio libraries contained nearly 70–80 Gbp of sequencing data, we randomly selected 50 Gbp of data for further analysis in order to obtain quantitatively comparable data to the Nanopore sequencing. This 50 Gbp of data was then aligned as per section 1.4.1 to the publicly available *B. napus* reference and also to the concatenated pseudo‐reference assembly, using NGMLR version 0.2.7 with default settings. NGMLR produced an un‐sorted SAM file as an output, which was converted to a sorted BAM file using Samtools version 1.9. Genomic variants were called using Sniffles (version 1.0.10) using the preset parameters.

#### Quality filtering of the predicted SV events for both ONT and PacBio datasets

We performed a very stringent quality filtering on the sniffles predicted SV events. Since the study was focused on small‐scale insertions or deletions, we removed all predicted translocations and duplications. Furthermore, it is nearly impossible to validate the authenticity of such SV events, as many may represent mis‐positioning of genomic fragments in the reference assembly, we only considered SV scored as 'PASS' by Sniffles and ignored those scored as 'UNRESOLVED'. Sniffles report SVs with both within‐alignment (AL) and split‐read (SR) information. AL‐type SV are usually small indels that can be spanned within a single alignment, whereas large or complex events lead to SR alignments (Sedlazeck *et al*., 2018). To ensure only the high‐confidence SV were selected, all SV which were not supported by a 'within‐alignment: AL' flag were discarded. This might lead to an under‐estimation and bias in the size distribution of the detectable SV. However, at this point of time the accuracy of publically available genome from *B. napus* is not high enough to distinguish large and complex SV events from assembly errors.

#### Comparison of SV calls between ONT and PacBio datasets for Express 617

To evaluate the impact of the sequencing technology on the general ability to accurately call small‐ to mid‐scale SV, we used SURVIVOR version 1.0.7 (Jeffares *et al*., 2017) to calculate overlaps between SV calls for ONT and PacBio datasets for the same genotype, Express 617. In a first step, quality‐filtered SV calls from the respective PacBio and ONT datasets were merged using 'SURVIVOR merge' using the settings 'Max distance between breakpoints 1000, Minimum number of supporting caller 1, Take the type into account 1, Take the strands of SVs into account −1, Estimate distance based on the size of SV −1, Minimum size of SVs to be taken into account −1'. The merged variant calling file (VCF) was then used to 'force call' the SV across both datasets using Sniffles version 1.0.10. These forced‐called SV were again merged into a single VCF using 'SURVIVOR merge' with 'Max distance between breakpoints 1000, Minimum number of supporting caller −1, Take the type into account 1, Take the strands of SVs into account −1, Estimate distance based on the size of SV −1, Minimum size of SVs to be taken into account −1'. Overlap was estimated by counting the number of SV that could be genotyped in both datasets.

### CNV calling

CNV detection was performed using the method published by Stein *et al*. (2017). Read depth for every nucleotide in the genome was calculated using the *genomecov *command in bedtools package v.2.20.1, using the alignment file from NGMLR as input. This read depth file was then used for estimating median coverage over 1000 bp blocks using an R script published in Stein *et al*. (2017). We then calculated median read coverage and standard deviation separately for every *B. napus* chromosome. Genomic segments with coverage higher than 1 and standard deviation above the median depth of the chromosome were defined as segmental duplications or CNV.

### Calculation of overlap between SV events and the gene models

Quality‐filtered SV events were overlapped with the gene models from Darmor*‐bzh* and also to the combined *B. rapa* and *B. oleracea* reference assemblies using bedtools intersect (Quinlan and Hall, 2009) using the default parameters. In order to calculate the genome‐wide frequency of SV events, we also overlapped the quality‐filtered SV with a bed file containing 1 Mbp windows for the entire genome assembly. The intersect file between the SV events and 1 Mbp windows for the entire genome assembly was then used for plotting the SV distribution along 19 *B. napus* chromosomes, using Circos (Krzywinski *et al*., 2009). Statistics including length and distribution of quality‐filtered SV from the 12 genotypes were calculated with SURVIVOR (Jeffares *et al*., 2017) and plotted with ggplot2 (Wickham, 2016).

### Construction of a maximum‐likelihood (ML) tree

SV events predicted for each of the 12 genotypes were merged into a single VCF. This combined VCF was then used to force call all the SV events across all 12 genotypes using Sniffles, resulting in a multi‐sample VCF. The multi‐sample VCF was then converted into PHYLIP format using an in house bash script and used as an input for IQ‐TREE version 1.6.12 (Nguyen *et al*., 2015). The best‐fit substitution model for the data was determined by IQ‐TREE ModelFinder (Kalyaanamoorthy *et al*., 2017) and used to construct a phylogenetic tree. The tree was then plotted with FigTree (http://tree.bio.ed.ac.uk/software/figtree/).

### Selection of SV events for PCR validation

We looked at two different agronomically interesting traits in order to prioritize the predicted SV events. Firstly, we analyzed the SV events that might contribute to *Verticillium longisporum* (VL) resistance, using a bi‐parental double‐haploid population derived from a cross between our sequencing panel genotypes Express 617 and R53. Two QTL were defined for VL resistance on chromosome C01 and C05 by Obermeier *et al*. (2013). We mainly focused on C05 QTL, as this was described to be the major genetic control for VL resistance. The genetic map used for identifying C05 QTL was based on SSR (simple sequence repeats) and AFLP (amplified fragment length polymorphism) markers. Therefore, in order to localize the physical position of the QTL on chromosome C05, we anchored the flanking SSR markers (BRMS030_210 and Na12C01_160) to the Darmor‐*bzh* version 4.1 assembly and identified a 4.3 Mbp (6 329 426 bp to 10 659 726 bp) region containing 606 genes. Thirty‐seven and 45 out of the 606 genes were found to contain SV in the form of insertions or deletions in Express 617 and R53, respectively. A total of 17 genes were found to be common among both the genotypes, so were dropped from the prioritized gene set. We further prioritized the candidate genes, if they were annotated as defence response or phenolpropanoid pathway genes. Secondly, we analyzed the SV located within the genes described to be involved in flowering‐time pathway in *B. napus* as described by Schiessl *et al*. (2017). Top prioritized SV were then visualized in IGV viewer (Robinson *et al*., 2017) and selected for PCR validation.

## Conflict of interest

The authors declare no conflicts of interest.

## Author contributions

HSC, HTL and RJS conceived the study. HSC, STNA and IAPP generated the Oxford Nanopore long‐read sequence data. JS, KL and LG contributed PacBio long‐read sequence data. SVS contributed Illumina sequence‐capture data. HSC, STNA and HTL conducted the experiments and analysed the data. PV performed SV validation. IG, CO, RJS and HTL provided ideas and suggestions for data analysis. HSC and RS drafted the manuscript.

## Supporting information


**Table S1** Read statistics for raw reads.
**Table S2** SV Length distribution compared to *B. napus* 4.1 reference assembly.
**Table S3** SV Length distribution compared to *B. rapa* plus *B. oleracea* pseudoassembly.
**Table S4** Average number of SV per megabase along each *B. napus* chromosome (compared to *B. napus* 4.1 reference assembly).
**Table S5** Average number of SV per megabase along each *B. napus* chromosome (compared to *B. rapa* plus *B. oleracea* pseudo assembly).
**Table S6** Average number of deletions per megabase along each *B. napus* chromosomes (compared to *B. napus* 4.1 reference assembly).
**Table S7** Average number of insertions per megabase along each *B. napus* chromosome (compared to *B. napus* 4.1 reference assembly).
**Table S8** Number of CNV in four representative *B. napus* accessions.
**Table S9** CNV calling for Express 617, R53, N99 and PAK85912.
**Table S10** Validation of 1.3 Kbp deletion in the promoter region of *BnFT.A02* (BnaA02g12130D) in ERANET‐ASSYST consortium diversity set.
**Table S11** Distribution of SV in exons and introns.
**Table S12** PCR primer sequences used for validation of SV events.

## Data Availability

Raw data from all 4 ONT libraries have been deposited to the NCBI short‐read archive under Bio project number PRJNA642096. The PacBio data from Song *et al*. (2020) are available under PRJNA546246. All variants detected in this study are available as a Supplementary Dataset at https://doi.org/10.5281/zenodo.3931391
